# Supplementary material for: Alloying and confinement effects on hierarchically nanoporous CuAu for efficient electrocatalytic semi-hydrogenation of terminal alkynes
Source: Nat Commun. 2024 Jul 17;15:5999. doi: 10.1038/s41467-024-50499-3 (PMC11252328; doi:10.1038/s41467-024-50499-3)
Supplement: Supplementary file 1 — Supplementary Information [file 41467_2024_50499_MOESM1_ESM.pdf]

# Alloying and Confinement effects on Hierarchically Nanoporous CuAu for Efficient Electrocatalytic Semi-Hydrogenation of Terminal Alkynes

Linghu Meng[1,5], Cheng-Wei Kao[2,5], Zhen Wang[1,5], Jun Ma[3], Peifeng Huang[3], Nan Zhao[4], Xin Zheng[4], Ming Peng[1], Ying-Rui Lu[2], Yongwen Tan[1*]

[1]College of Materials Science and Engineering, State Key Laboratory of Advanced Design and Manufacturing Technology for Vehicle Body, Hunan University, Changsha, Hunan 410082, China

[2]National Synchrotron Radiation Research Center, Hsinchu, 300092, Taiwan

[3]College of Mechanical and vehicle Engineering, Hunan University, Changsha, Hunan 410082, China

[4]Electrical Power Research Institute of Yunnan Power Grid Co. Ltd, North China Electric Power, Kunming, Yunnan, 650217, China

[5]Linghu Meng, Cheng-Wei Kao and Zhen Wang contributed equally to this work

[*]e-mail: tanyw@hnu.edu.cn

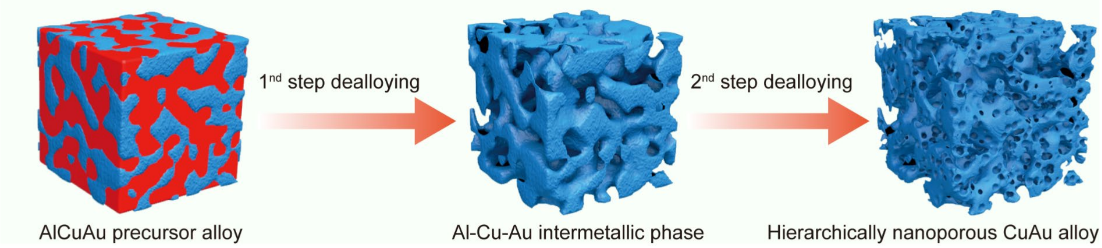


**Supplementary Fig. 1| Schematic illustration of the preparation of Hnp-$Cu_{100-x}Au_x$.** Illustration of the synthetic process of Hnp-$Cu_{100-x}Au_x$.

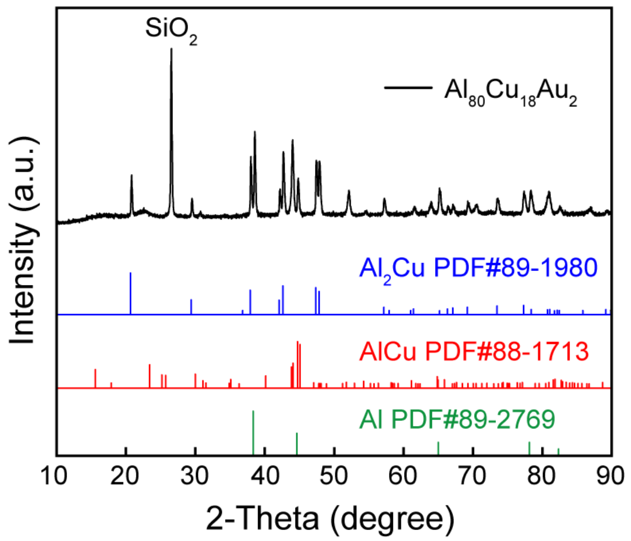


**Supplementary Fig. 2| XRD characterizations of $Al_{80}Cu_{18}Au_2$.** XRD patterns of the $Al_{80}Cu_{18}Au_2$.

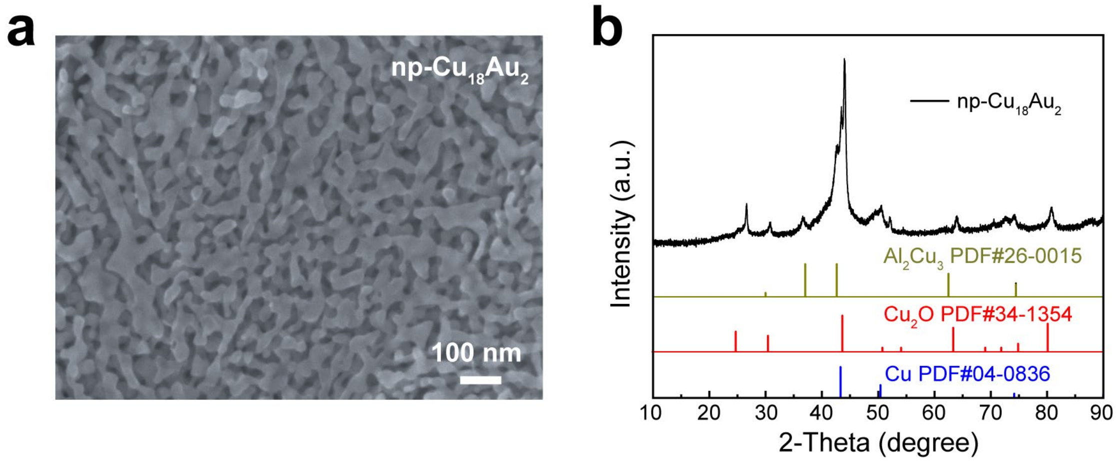


**Supplementary Fig. 3| SEM and XRD characterizations of np-$Cu_{18}Au_2$.** (a) SEM images of the np-$Cu_{18}Au_2$, (b) XRD patterns of the np-$Cu_{18}Au_2$.

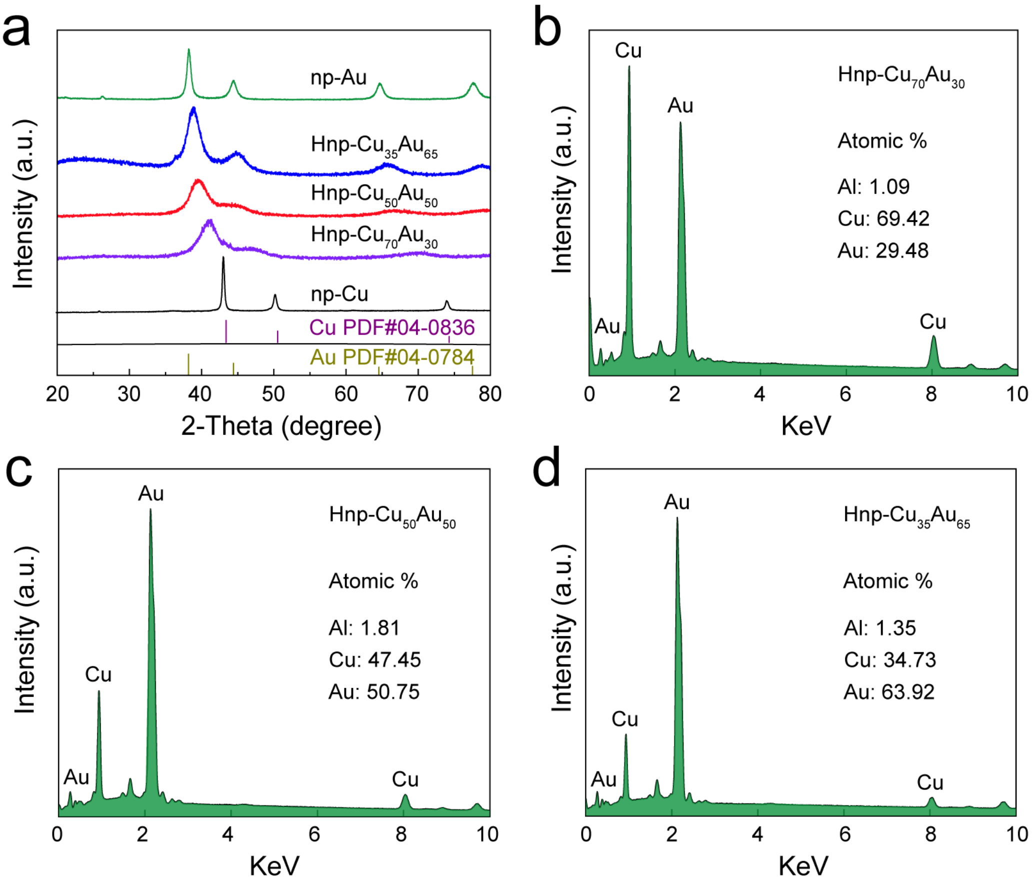


**Supplementary Fig. 4| XRD and EDS characterizations.** (a) XRD patterns of np-Cu, Hnp-$Cu_{70}Au_{30}$, Hnp-$Cu_{50}Au_{50}$, Hnp-$Cu_{35}Au_{65}$ and np-Au. (b-d) EDS spectra of the Hnp-$Cu_{70}Au_{30}$, Hnp-$Cu_{50}Au_{50}$ and Hnp-$Cu_{35}Au_{65}$.

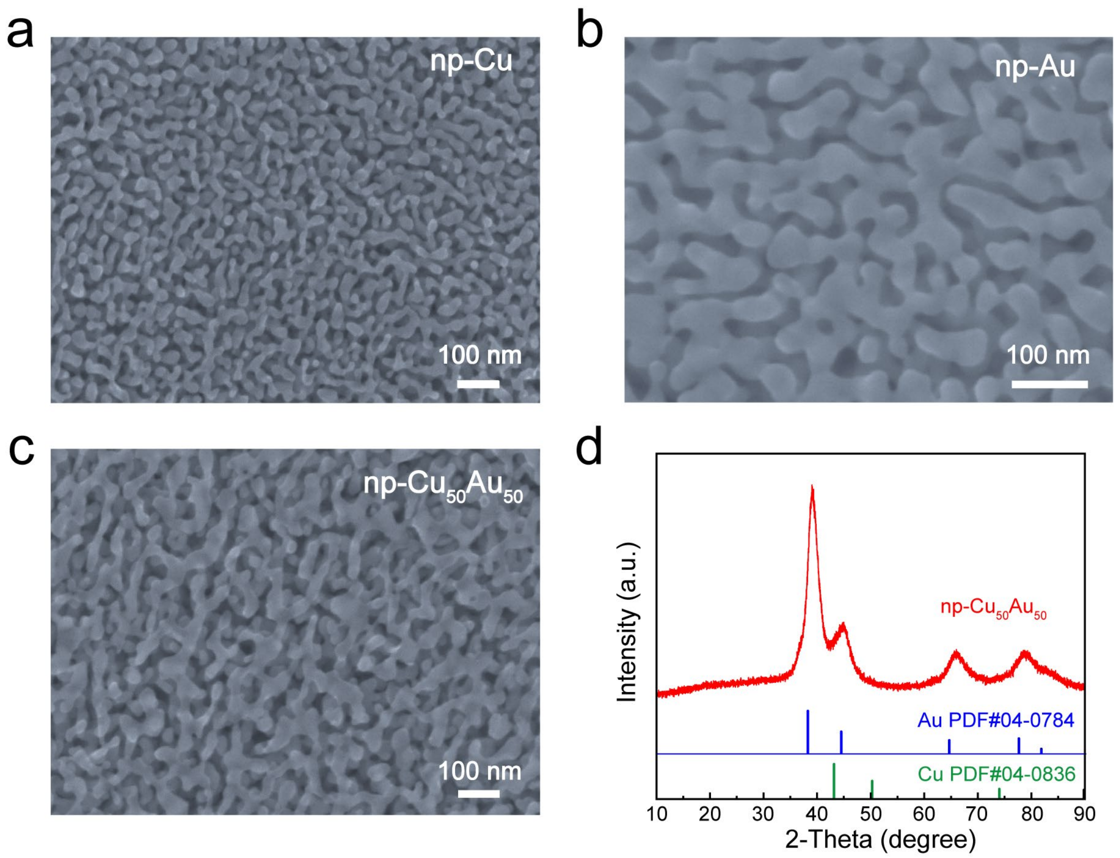


**Supplementary Fig. 5| SEM and XRD characterizations.** (a-c) SEM images of (a) np-Cu, (b) np-Au and (c) np-$Cu_{50}Au_{50}$. (d) XRD patterns of the np-$Cu_{50}Au_{50}$.

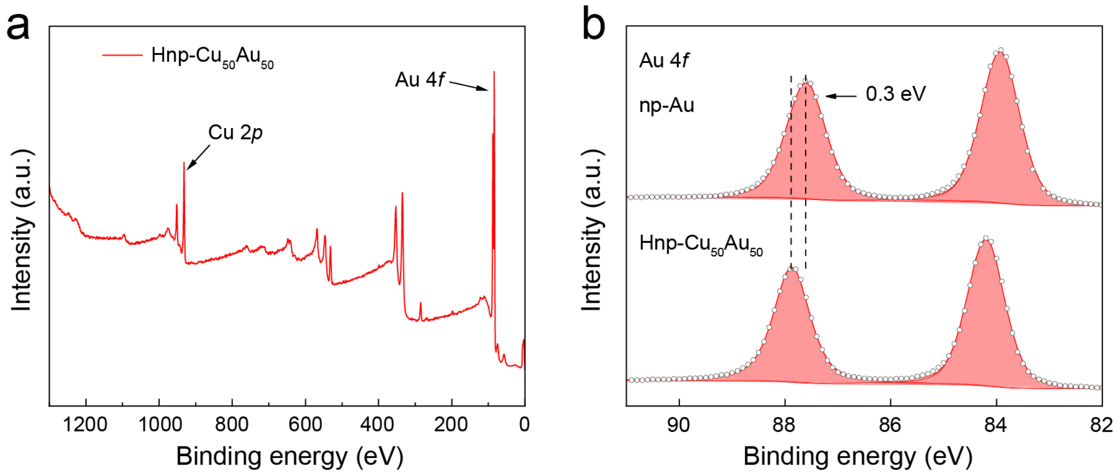


**Supplementary Fig. 6| XPS characterizations.** (a) XPS full spectra of Hnp-$Cu_{50}Au_{50}$, (b) XPS spectra of Hnp-$Cu_{50}Au_{50}$ and np-Au in Au 4*f*.

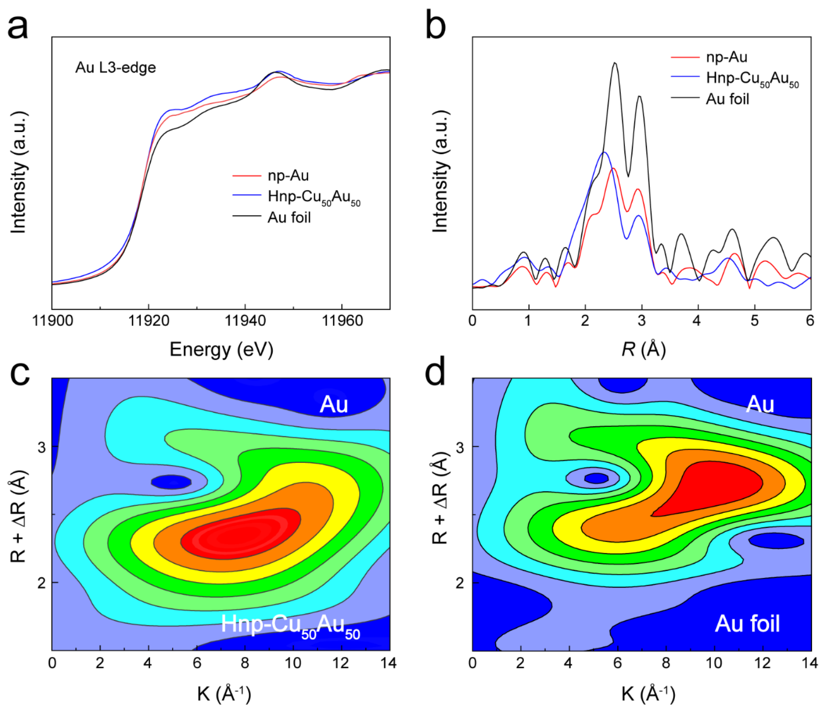


**Supplementary Fig. 7| Synchrotron radiation characterization of the np-Au and Hnp-$Cu_{50}Au_{50}$.** (a) Normalized XANES at the Au L3-edge of Hnp-$Cu_{50}Au_{50}$, np-Au, Au foil. (b) The corresponding FT-EXAFS spectra of the Au L3-edge derived from (a). (c, d) WT of Au L3-edge EXAFS spectra of Hnp-$Cu_{50}Au_{50}$ and Au foil.

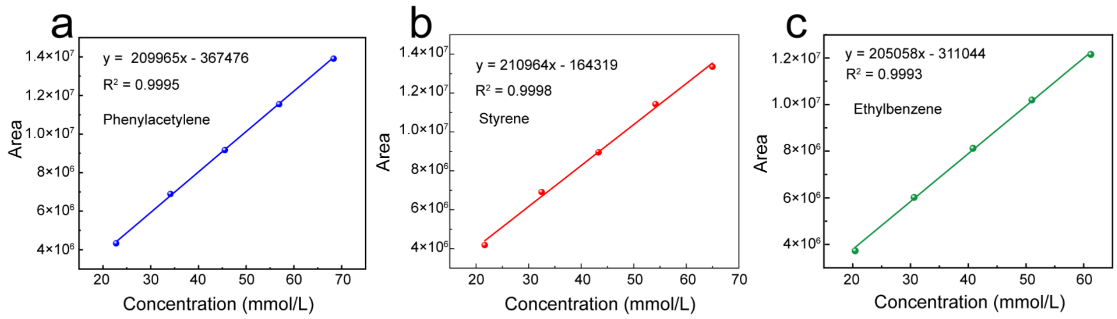


**Supplementary Fig. 8| The GC-MS standard curves for quantitative analysis.** (a) Phenylacetylene, (b) Styrene and (c) Ethylbenzene.

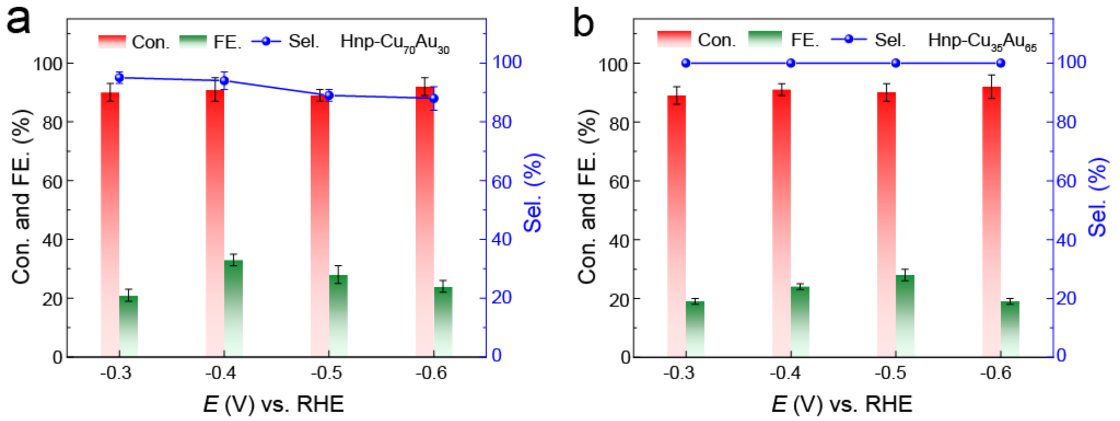


**Supplementary Fig. 9| Electrocatalytic performance of Hnp-$Cu_{70}Au_{30}$ and Hnp-$Cu_{35}Au_{65}$ for alkyne semi-hydrogenation.** Potential-dependent conversions of phenylacetylene, selectivity and FE of styrene over (a) Hnp-$Cu_{70}Au_{30}$, (b) Hnp-$Cu_{35}Au_{65}$ in 1 M KOH. The error bars represent the standard deviation for at least three independent measurements.

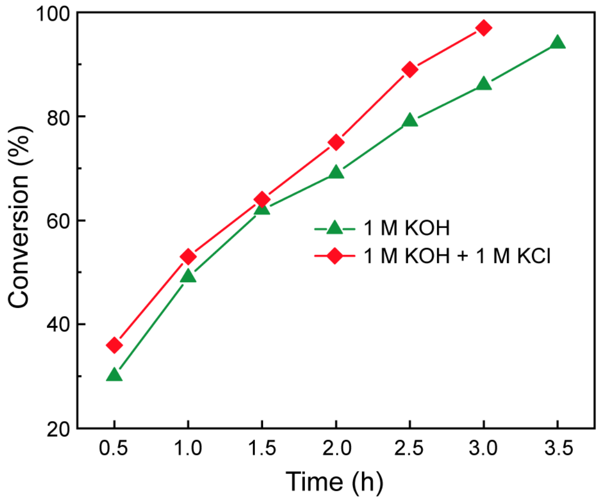


**Supplementary Fig. 10| Time-dependent conversion of phenylacetylene in 1 M KOH with and without 1 M KCl.** Time-dependent conversion change of phenylacetylene with and without 1 M KCl over Hnp-$Cu_{50}Au_{50}$ at -0.6 V versus RHE.

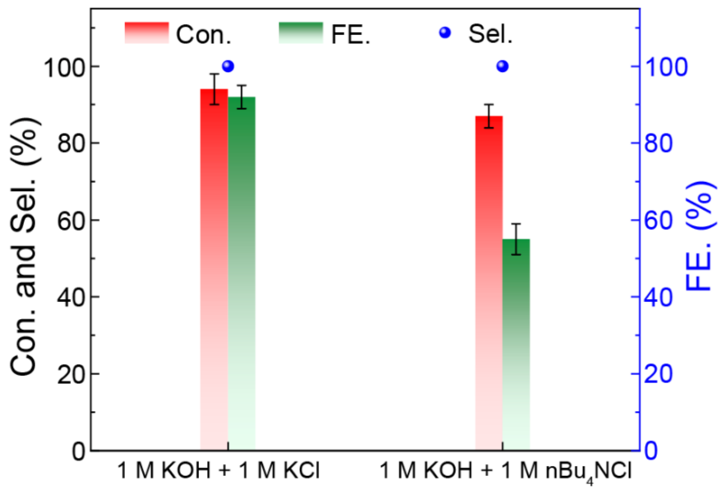


**Supplementary Fig. 11| Electrocatalytic performance for alkyne semi-hydrogenation in 1 M KOH with 1 M KCl and 1 M $nBu_4NCl$.** Conversions of phenylacetylene, selectivity and FE of styrene over Hnp-$Cu_{50}Au_{50}$ in 1 M KOH + 1 M KCl and 1 M KOH + 1 M $nBu_4NCl$. The error bars represent the standard deviation for at least three independent measurements.

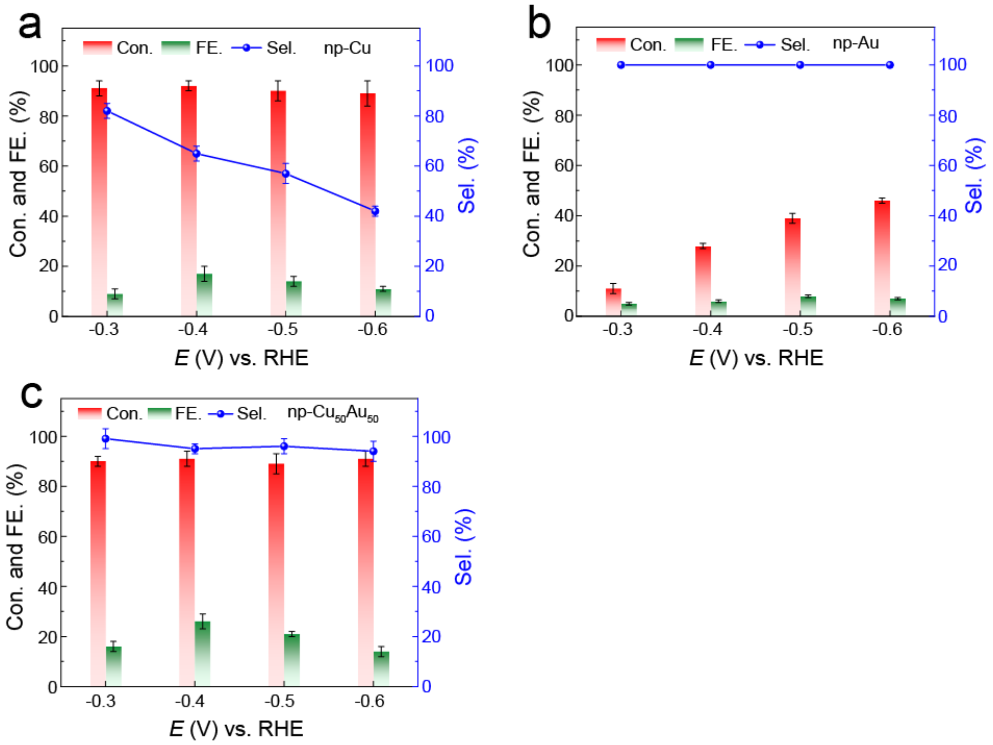


**Supplementary Fig. 12| Electrocatalytic performance of catalysts for alkyne semi-hydrogenation.** Potential-dependent conversions of phenylacetylene, selectivity and FE of styrene in 1 M KOH + 1 M KCl over (a) np-Cu, (b) np-Au and (c) np-$Cu_{50}Au_{50}$. The error bars represent the standard deviation for at least three independent measurements.

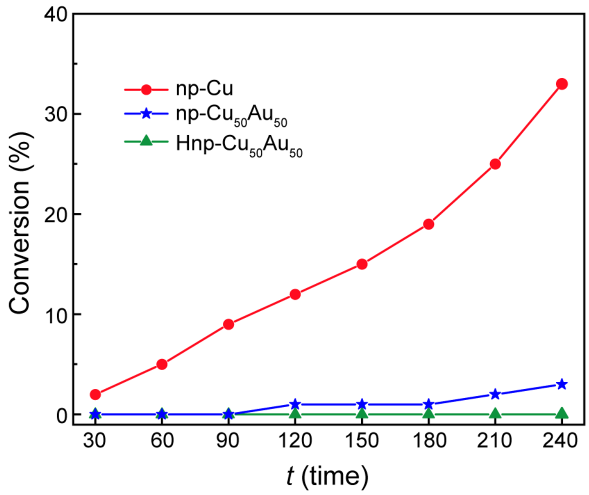


**Supplementary Fig. 13| Time-dependent conversion of styrene.** Time-dependent conversions of styrene over np-Cu, np-$Cu_{50}Au_{50}$, and Hnp-$Cu_{50}Au_{50}$.

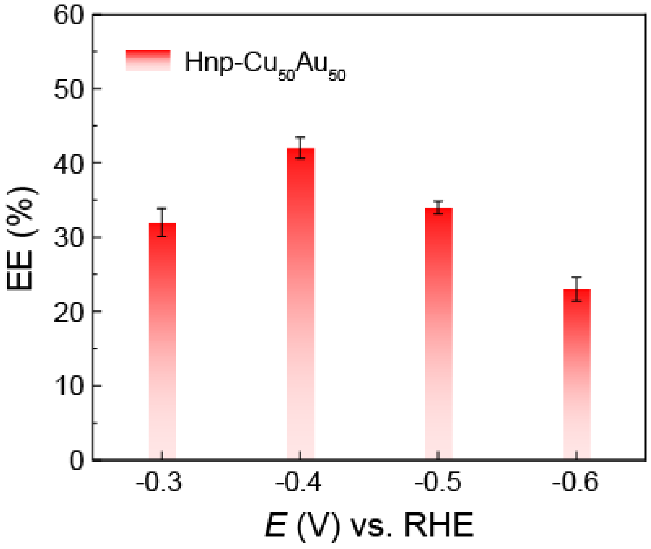


**Supplementary Fig. 14| The energy efficiency of alkyne semi-hydrogenation.** The energy efficiency of Hnp-$Cu_{50}Au_{50}$ for semi-hydrogenation of phenylacetylene at different applied potentials.

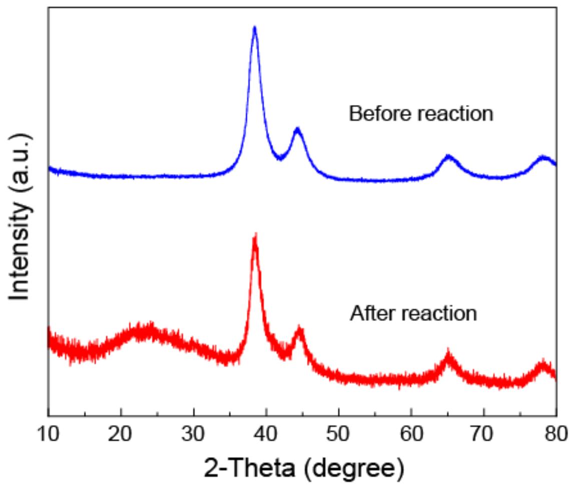


**Supplementary Fig. 15| XRD characterizations of Hnp-$Cu_{50}Au_{50}$.** XRD patterns of Hnp-$Cu_{50}Au_{50}$ before and after reaction.

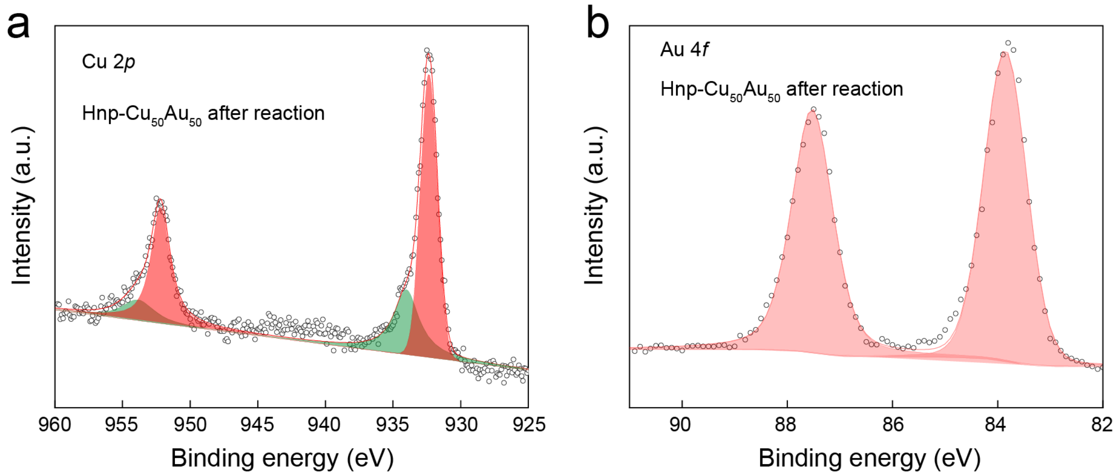


**Supplementary Fig. 16| XPS Characterizations of the Hnp-$Cu_{50}Au_{50}$ after reaction.**

(a) Cu 2*p* XPS spectra. (b) Au 4*f* XPS spectra.

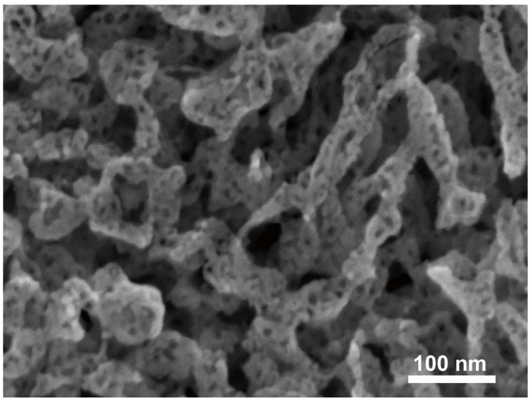


**Supplementary Fig. 17| SEM characterizations of Hnp-$Cu_{50}Au_{50}$ after reaction.**

SEM image of Hnp-$Cu_{50}Au_{50}$ alloy after reaction.

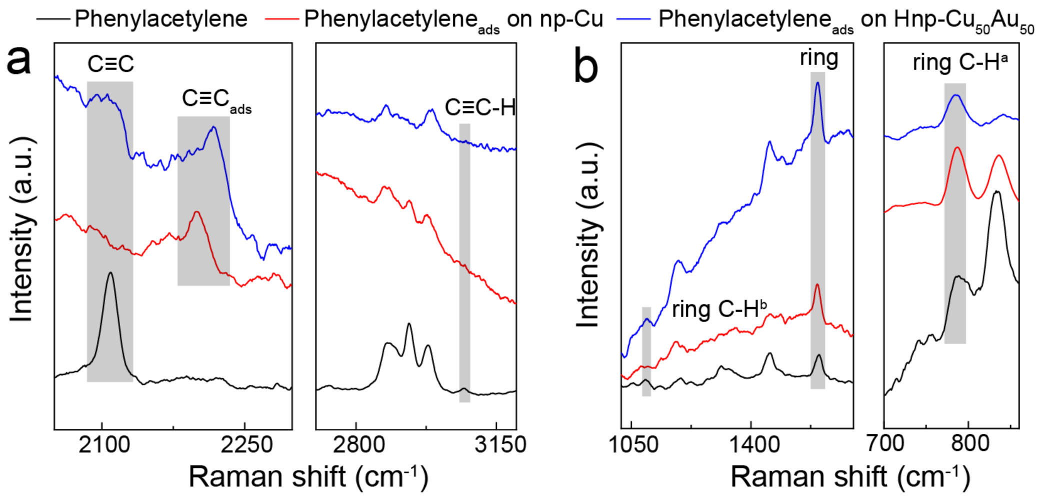


**Supplementary Fig. 18| In situ Raman spectra for the electrocatalytic hydrogenation of phenylacetylene.** In situ Raman tests in a mixed 1.0 M KOH/Diox solution for electrocatalytic hydrogenation of phenylacetylene at −0.4 V vs. RHE over (a) np-Cu, (b) Hnp-$Cu_{50}Au_{50}$.

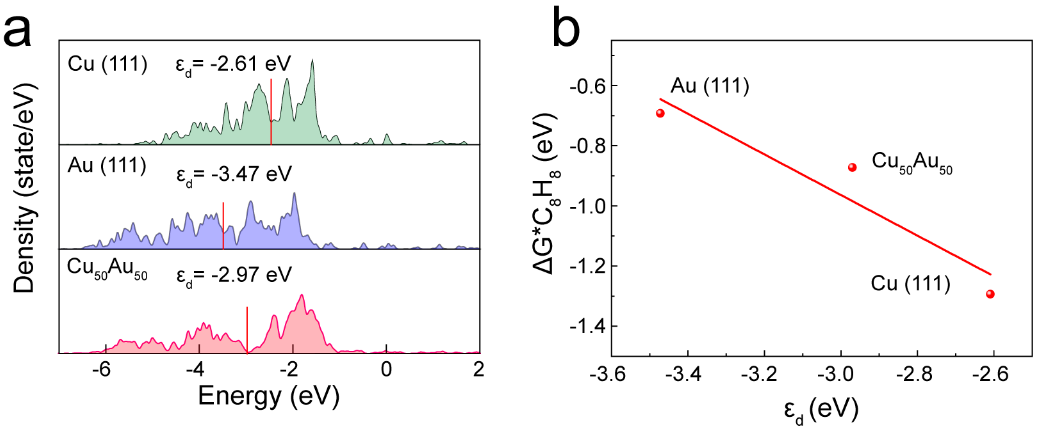


**Supplementary Fig. 19| DFT calculations.** (a) PDOS of Cu (111), Au (111) and $Cu_{50}Au_{50}$, (b) the relationship between d-band center and free energy of $*C_8H_8$ adsorption.

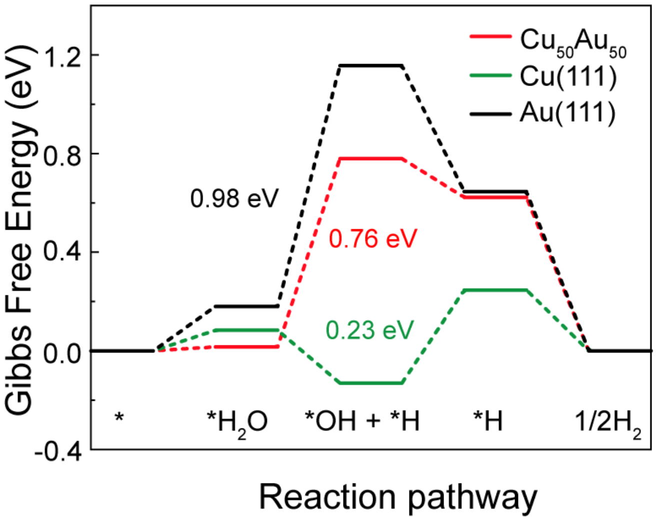


**Supplementary Fig. 20| Free energy diagram for water-splitting process.** Free energy diagram for water-splitting process over Cu (111), Au (111), and $Cu_{50}Au_{50}$.

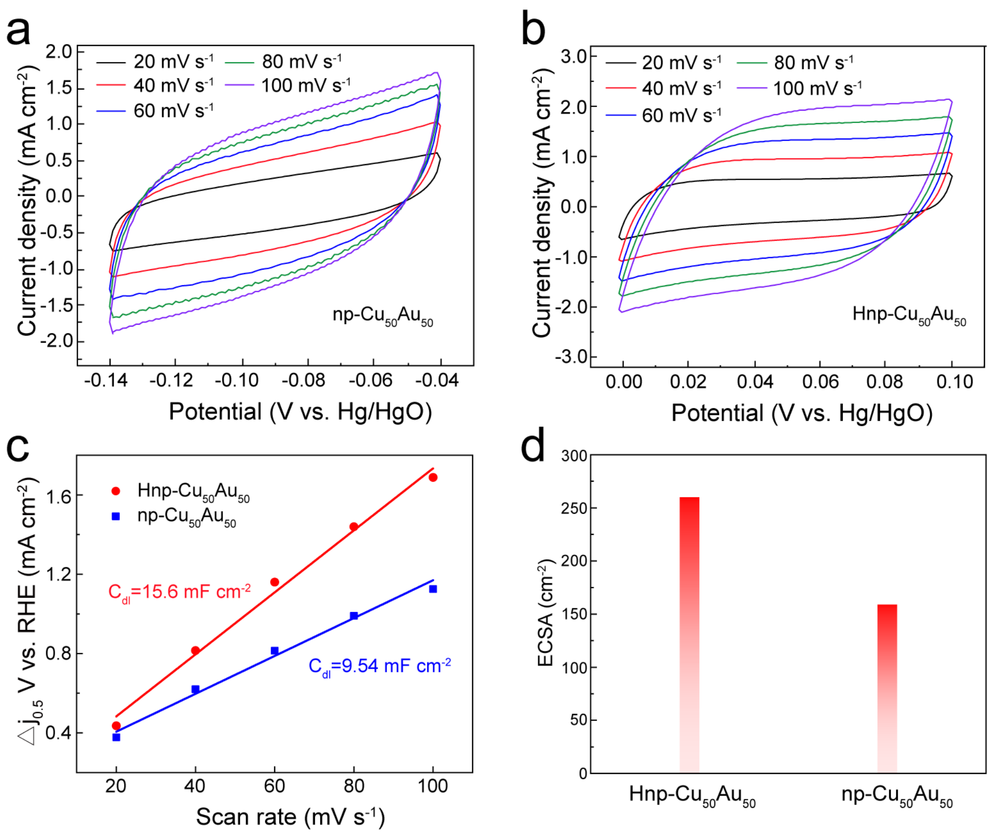


**Supplementary Fig. 21| Double-layer capacitance analyses.** (a, b) CVs of np-$Cu_{50}Au_{50}$ (a) and Hnp-$Cu_{50}Au_{50}$ (b). These CVs were performed at various scan rates 20, 40, 60, 80, and 100 mV $s^{-1}$. (c) The plots of current densities against scan rates. $\Delta j$ is the difference between anodic and cathodic current densities at a same potential (0.05 V. vs. RHE). (d) The electrochemically active surface areas (ECSAs) of Hnp-$Cu_{50}Au_{50}$ and np-$Cu_{50}Au_{50}$.

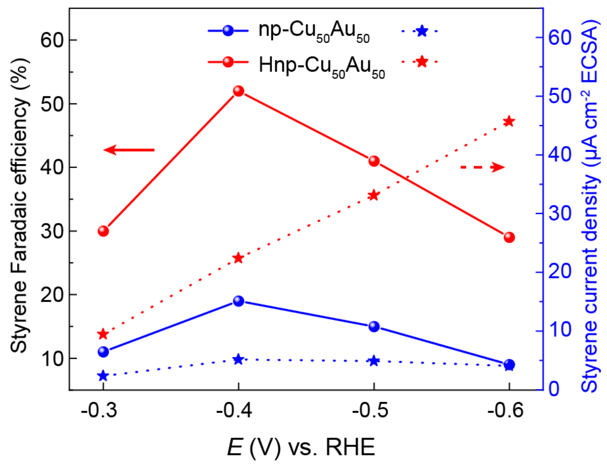


**Supplementary Fig. 22| The comparison of styrene FE and ECSA-normalized styrene current density.** The styrene FE and ECSA-normalized styrene current density comparison between Hnp-$Cu_{50}Au_{50}$ and np-$Cu_{50}Au_{50}$ in 1 M KOH.

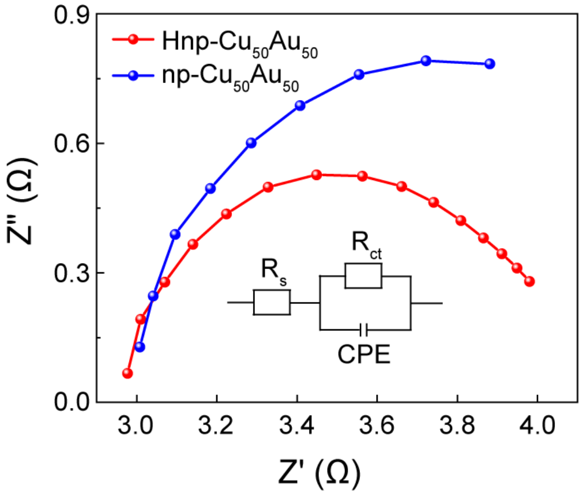


**Supplementary Fig. 23| Electrochemical impedance spectroscopy analyses.** Nyquist plots of Hnp-$Cu_{50}Au_{50}$ and np-$Cu_{50}Au_{50}$ at -0.4 V vs. RHE. Inset: equivalent circuit for EIS fitting.

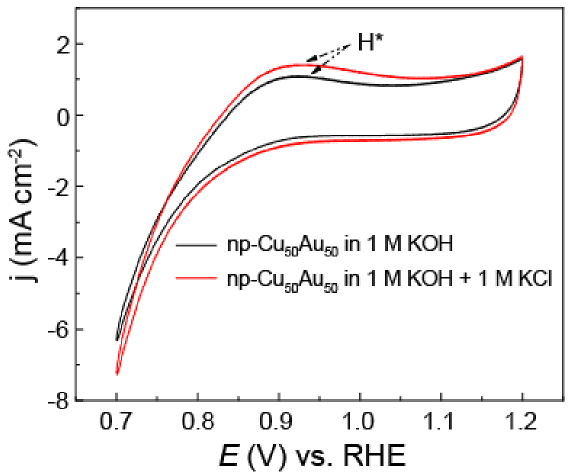


**Supplementary Fig. 24| CV curves of np-$Cu_{50}Au_{50}$ in 1 M KOH with or without 1 M KCl.** CV curves of np-$Cu_{50}Au_{50}$ in 1 M KOH and 1 M KOH + 1 M KCl with a scan rate of 100 mV $s^{-1}$.

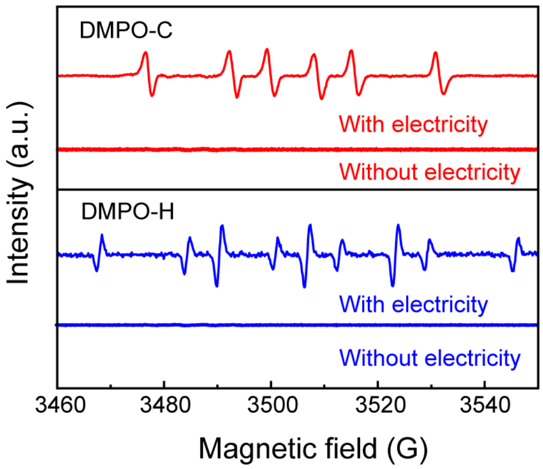


**Supplementary Fig. 25| Quasi-in situ EPR tests.** Quasi-in situ EPR trapping for carbon and hydrogen radicals over Hnp-$Cu_{50}Au_{50}$ at -0.4 V vs. RHE.

a

| | Energy (eV) | ΔG(298.15) (eV) | G (eV) |
|---|---|---|---|
| $H_2$ (g) | -6.76028 | -0.046624 | -6.80591 |
| $C_8H_6$ | -92.0194 | 2.084939 | -89.9345 |
| $C_8H_8$ | -100.899 | 2.681275 | -98.2175 |
| $H_2O$ (l) | | | -14.22 |

b

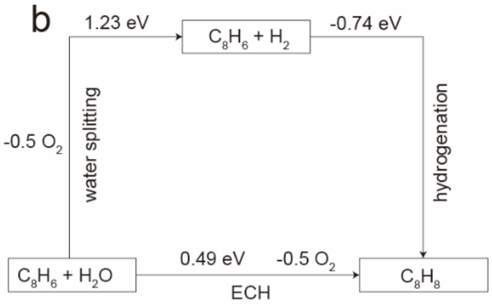


**Supplementary Fig. 26| Standard equilibrium potential of phenylacetylene to styrene.** (a) Standard Gibbs free energies of $H_2$(g), $C_8H_6$, $C_8H_8$ and $H_2O$(l). (b) The standard equilibrium potential of phenylacetylene-to-styrene conversion routes.

**Supplementary Table 1**. Summary of conversion (Con), selectivity (Sel.) and Faradaic efficiency (FE) values of the reported catalysts in the electrocatalytic semi-hydrogenation of alkynes to alkynes.

| Catalyst | Reactant | Product | Con (%) | Sel (%) | FE (%) | Reference |
|---|---|---|---|---|---|---|
| Hnp-$Cu_{50}Au_{50}$ | 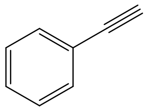 | 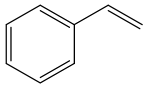 | 94 | 100 | 92 | This work |
| Pt/$W_2C$-7.4 | 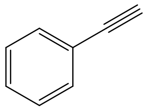 | 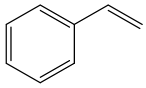 | 32 | 95 | 53 | [1] |
| PdFe | 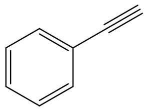 | 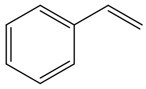 | 96.1 | 92.9 | — | [2] |
| PdCu | 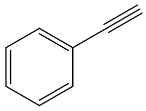 | 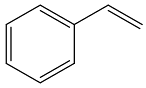 | 97.4 | 93.0 | 9.5 | [3] |
| PdP | 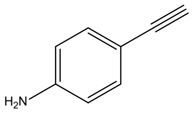 | 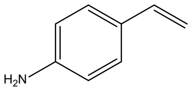 | 92 | 98 | 78 | [4] |
| $Pd_2N$ | 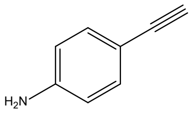 | 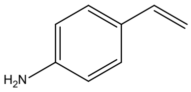 | 95.7 | 97.8 | 72.4 | [5] |
| Pd@ArS-$Pd_4S$ | 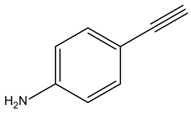 | 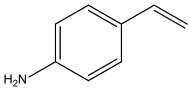 | 97 | 96 | 75 | [6] |
| Cu-S | 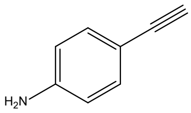 | 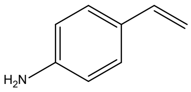 | 99 | 99 | 10 | [7] |
| $PdS_x$ | 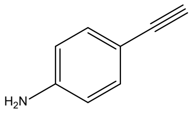 | 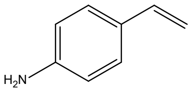 | 67 | 98 | 28 | [8] |

**Supplementary Table 2**. Substrate scope for electrocatalytic semi-hydrogenation of alkynes with $H_2O$ over a Hnp-$Cu_{50}Au_{50}$ cathode.

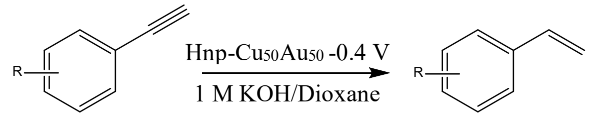


| | | |
|---|---|---|
| 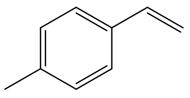 | 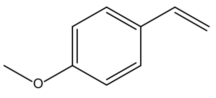 | 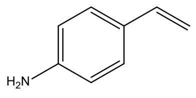 |
| 95% (94%) | 93% (96%) | 98% (100%) |
| 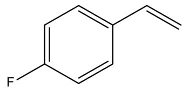 | 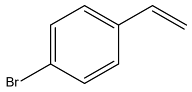 | 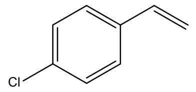 |
| 91% (100%) | 88% (93%) | 94% (97%) |

Reaction conditions: alkynes substrates (1 mmol), Hnp-$Cu_{50}Au_{50}$ (working area: 1 $cm^2$), 1 M KOH (Dioxane/$H_2O$), room temperature, -0.4 V vs. RHE. Conversion yields were reported, and the data in parentheses were the alkene selectivity.

## Supplementary References


1. Xiu L. et al. Enrichment of polarized alkynes over negatively charged Pt for efficient electrocatalytic semihydrogenation. *ACS Catal*. **14**, 2173−2180 (2024).

2. Zhu, K. et al. Unraveling the role of interfacial water structure in electrochemical semihydrogenation of alkynes. *ACS Catal.* **12**, 4840−4847 (2022).

3. Xu, X. et al. Regulating the interfacial water structure by tensile strain to boost electrochemical semi-hydrogenation of alkynes. *Inorg. Chem. Front.* **9**, 3444 (2022)

4. Wu, Y., Liu, C., Wang, C., Lu, S. & Zhang, B. Selective transfer semihydrogenation of alkynes with $H_2O$ ($D_2O$) as the H (D) source over a Pd-P cathode. *Angew. Chem. Int. Ed.* **59**, 21170–21175 (2020).

5. Song, Z. et al. Interstitial modification of palladium nanocubes with nitrogen atoms promotes aqueous electrocatalytic alkyne semihydrogenation. *ACS Mater. Lett*. **5**, 3068−3073 (2023).

6. Gao, Y. et al. Field-induced reagent concentration and sulfur adsorption enable efficient electrocatalytic semihydrogenation of alkynes. *Sci. Adv*. **8**, eabm9477 (2022).

7. Wu, Y. et al. Converting copper sulfide to copper with surface sulfur for electrocatalytic alkyne semihydrogenation with water. *Nat. Commun.* **12**, 3881 (2021).

8. Li, H. et al. σ-alkynyl adsorption enables electrocatalytic semihydrogenation of terminal alkynes with easy-reducible/passivated groups over amorphous $PdS_x$ nanocapsules. *J. Am. Chem. Soc.* **144**, 19456−19465 (2022).
